# Defining neurovascular ecosystem states with single-cell multi-omics and machine learning: insights into cerebrovascular remodeling in the tumor context

**DOI:** 10.3389/fcell.2026.1846929

**Published:** 2026-06-03

**Authors:** Dongsheng Kong, Yakun Chen, Wenwen Wang, Zhizhen Qin, Chenxuan Yang, Zhao Gao, Shengli Guo, Zhe Xue

**Affiliations:** Department of Neurosurgery, The First Medical Center, Chinese PLA General Hospital, Beijing, China

**Keywords:** brain tumor, cerebrovascular remodeling, interpretable machine learning, neurovascular ecosystem, single-cell multi-omics

## Abstract

**Objective:**

To define neurovascular ecosystem states within the tumor microenvironment and elucidate the regulatory basis of cerebrovascular remodeling through single-cell multi-omics and interpretable machine learning.

**Methods:**

An orthotopic murine brain tumor model was established. Single-cell RNA sequencing (scRNA-seq), single-cell assay for transposase-accessible chromatin sequencing (scATAC-seq), regional tissue validation, and integrative computational analyses were performed to characterize cellular heterogeneity, regulatory programs, and state transitions during tumor progression.

**Results:**

Tumor progression induced coordinated reprogramming across endothelial, mural, glial, and myeloid compartments. Trajectory inference modeled a progressive shift of endothelial cells from barrier-maintaining states toward angiogenic and inflammatory phenotypes, while mural cells exhibited transcriptional signatures indicative of matrix-remodeling adaptation. Extracellular matrix remodeling emerged as a shared neurovascular program with pronounced spatial heterogeneity. Epigenomic profiling further supported stable regulatory transitions, and interpretable machine learning prioritized key determinants associated with angiogenesis, barrier dysfunction, inflammation, hypoxic adaptation, and perivascular remodeling.

**Conclusion:**

Cerebrovascular remodeling in brain tumors reflects a dynamic, multicellular reorganization of the neurovascular ecosystem rather than an isolated vascular abnormality. These findings provide a systems-level framework for understanding tumor-associated vascular remodeling and its regulatory basis.

## 1 Introduction

During the progression of malignant tumors in the nervous system, particularly gliomas, the remodeling of tumor cells within the surrounding microenvironment constitutes a complex and systematic evolutionary process. Traditional pathological perspectives often focus on the proliferation of tumor cells themselves, but increasing evidence indicates that neurovascular units composed of endothelial cells, pericytes, astrocytes, and immune cells undergo profound phenotypic remodeling during tumor evolution (Reid et al., 2025; Greenwald et al., 2024). This remodeling transcends the scope of simple angiogenesis, manifesting as a cross-cellular type co-evolution that forms a pathological ecosystem supporting tumor growth and infiltration (Xie et al., 2024). Within this ecosystem, structural integrity of vascular walls gradually deteriorates, tight junction proteins of the blood-brain barrier are downregulated, pericytes detach from the basement membrane and transform into pro-inflammatory fibroblast-like cells, while astrocytes exhibit significant reactive proliferation (Schot et al., 2024). These changes do not occur in isolation but are interconnected through complex paracrine signaling and biomechanical signals, collectively constructing a dynamically coupled neurovascular microenvironment.

Although the academic community has gained certain insights into the molecular mechanisms of tumor angiogenesis, traditional whole-body transcriptome sequencing technologies exhibit significant limitations in revealing neurovascular unit heterogeneity (Kang et al., 2024). Due to the low proportion of vascular components in brain tissue, signal averaging effects in whole-body transcriptomics often mask the characteristics of rare key cell subpopulations, particularly those at the forefront of remodeling such as tip cells and stem-like vascular progenitor cells. Additionally, the cerebrovascular system demonstrates high regional specificity in both anatomy and function, with notable differences in gene expression profiles between vascular cells in tumor core regions and peripheral infiltrated areas (Liu et al., 2025). The emergence of single-cell transcriptome sequencing technologies has provided a critical tool for addressing this challenge, enabling researchers to precisely map molecular profiles of the cerebrovascular system at single-cell resolution (Fu et al., 2025). By capturing transcriptional state changes in cells at different spatiotemporal nodes, we can identify functionally specialized subpopulations induced by tumor stimulation, thereby elucidating how tumors achieve pathological vascular remodeling through hijacking normal vascular development pathways.

When deciphering the deep regulatory mechanisms of vascular remodeling, single-dimensional transcriptomic data often fail to fully reveal the driving factors underlying cellular phenotypes. Cellular state transitions are not only regulated by immediate transcriptional levels but also profoundly influenced by epigenetic features such as chromatin accessibility. The integrated application of single-cell multi-omics technologies—particularly the combination of transcriptomics and chromatin accessibility profiling—provides a comprehensive perspective spanning from epigenomic blueprints to transcriptional execution (Li et al., 2024). Under tumor-induced stress conditions, vascular cells often enter a pre-stimulated chromatin state before significant gene expression alterations occur. This epigenetic preprogramming determines cellular response logic in environmental stimuli. However, high-dimensional data generated by multi-omics approaches also pose challenges to traditional bioinformatics analysis. Machine learning algorithms demonstrate remarkable advantages in processing nonlinear relationships and complex pattern recognition (Ordóñez-Rubiano et al., 2025). Rather than serving as mere descriptive labels, we define ‘neurovascular ecosystem states’ as cross-modal functional units characterized by the integrated synchronization of transcriptional outputs and chromatin accessibility landscapes. By constructing supervised learning models on these multi-dimensional feature vectors, we transition from qualitative clustering to an operational framework that achieves higher explanatory precision in identifying the stable regulatory programs that govern transitions between healthy and pathological niches.

This study employs a mouse orthotopic brain tumor model to investigate the systems-level logic of cerebrovascular remodeling. Rather than aiming to construct exhaustive standalone atlases or develop novel computational algorithms, we utilize single-cell multi-omics, regional partitioning, and interpretable machine learning collectively as an integrated, hypothesis-generating framework. By synthesizing these modalities, we aim to elucidate core cellular subpopulations and their associated molecular programs during tumor-induced adaptation (Kojima et al., 2024; Pfau et al., 2024). The research focuses on revealing how different cellular components achieve functional coupling through dynamic cell communication networks, as well as how chromatin remodeling confers vascular cell adaptability within the tumor microenvironment. While individual phenomena such as tumor angiogenesis and barrier breakdown are well-documented, this study seeks to demonstrate how these distinct processes are spatiotemporally coupled through shared epigenetic priming. Moving beyond descriptive characterization, we leverage interpretable machine learning to quantitatively prioritize regulatory dependencies, thereby identifying key network hubs—rather than isolated genes—that govern the transition between homeostatic and pathological niches.

## 2 Methods

### 2.1 Experimental design and murine brain tumor model

This study is an exploratory mechanistic experimental investigation based on a mouse *in situ* brain tumor model, aiming to systematically elucidate the dynamic evolutionary characteristics of the neurovascular ecosystem state under tumor conditions and to reveal the key molecular basis of cerebrovascular remodeling from the perspective of integrating single-cell multi-omics and machine learning. The study adopts a phased and hierarchical design to ensure both the capture of continuous changes in the neurovascular microenvironment during tumor progression and the alignment of correlations between single-cell sequencing, epigenomic analysis, and histological validation. The overall experimental protocol, animal grouping, sampling timing, and tissue allocation strategy are detailed in Table 1.

**TABLE 1:** Experimental design, animal grouping, and tissue collection strategy.

Group	Time point	n	Condition	Tissue allocation	Main analyses
Control	Day 0	10	Normal C57BL/6 mice without tumor implantation	3 for scRNA-seq and scATAC-seq; 7 for histology and validation	Baseline atlas construction and neurovascular reference profiling
Early tumor	Day 7	10	Orthotopic GL261-bearing mice	3 for scRNA-seq and scATAC-seq; 7 for histology and validation	Early neurovascular remodeling analysis
Intermediate tumor	Day 14	10	Orthotopic GL261-bearing mice	3 for scRNA-seq and scATAC-seq; 7 for histology and validation	Ecosystem state transition and regulatory analysis
Advanced tumor	Day 21	10	Orthotopic GL261-bearing mice	3 for scRNA-seq and scATAC-seq; 7 for histology and validation	Advanced remodeling and machine learning-based state stratification

Experimental animals were selected as SPF-grade C57BL/6 mice with uniform age, body weight range, and housing conditions to minimize interference from genetic background and environmental factors on the brain microenvironmental state. The *in situ* glioma modeling strategy was employed, wherein GL261 cells were stereotaxically inoculated into the mouse brain to establish a representative tumor-associated cerebrovascular remodeling model. Compared to subcutaneous tumorigenesis models, the *in situ* model more accurately simulates pathological processes during brain tumor growth, including blood-brain barrier disruption, abnormal angiogenesis, pericyte response, glial activation, and immune cell infiltration. Therefore, it is more suitable for studying neurovascular ecological remodeling in the tumor microenvironment.

To elucidate the temporal changes in neurovascular status during brain tumor progression, this study established four experimental groups: normal control group, early-stage tumor group, intermediate-stage group, and advanced-stage group, with 10 mice included in each group. The normal control group did not undergo tumor inoculation to provide a baseline neurovascular reference background from steady-state brain tissue. Tumor groups were sampled on days 7, 14, and 21 post-modeling, corresponding to the early adaptation phase, progressive remodeling phase, and advanced-stage significant remodeling phase of tumor progression, respectively. This grouping approach avoids the limitations of cross-sectional comparisons based on a single endpoint, facilitating dynamic identification of the formation, deviation, and transformation of neurovascular ecosystem states under varying tumor loads.

Regarding sample allocation, three out of every ten animals per group were designated for single-cell transcriptome sequencing and single-cell chromatin accessibility sequencing to ensure independent biological replicates in multi-omics data. The remaining seven animals were allocated for histological analysis, immunofluorescence assays, and related validation experiments to support morphological and protein expression-level confirmation of single-cell results. This design addresses both the sample quality and freshness requirements of high-throughput omics analysis, as well as the spatial structure and histopathological evidence demands in mechanistic studies, thereby enhancing the reliability and interpretability of overall research conclusions.

### 2.2 Tissue dissociation and single-cell multi-omics library preparation

After euthanizing the mice at the scheduled time point, the brain tissue was perfused with pre-cooled phosphate buffer to minimize the impact of residual blood in the brain on subsequent analyses. The brain tissue was then rapidly isolated, and the tumor-affected hemisphere was excised under ice conditions while preserving the tumor region and adjacent brain tissue as much as possible. All samples for single-cell sequencing were processed under low-temperature conditions to ensure cellular viability and molecular information stability.

Fresh brain tissue was minced and processed using mechanical dispersion combined with enzymatic digestion to prepare a single-cell suspension. After digestion, the sample was filtered through a 70 μm filter screen followed by red blood cell lysis and cell debris removal. Cell viability was assessed via trypan blue staining, with only samples meeting quality standards being selected for subsequent library construction. Samples intended for single-cell transcriptome sequencing were adjusted to optimal concentration levels before undergoing microdroplet-based single-cell capture and library assembly, with each sample targeting approximately 5,000 to 10,000 cells.

Single-cell chromatin accessibility sequencing utilizes nuclear suspensions prepared from samples of the same batch. After mild tissue lysis to release nuclei, the cells are filtered and purified for counting and quality assessment, followed by transposon reaction and library construction. The target capture quantity per sample is controlled at approximately 5,000 to 10,000 nuclei. All libraries undergo fragment distribution and concentration testing, with high-throughput sequencing initiated only after passing quality control.

To minimize batch bias, this study adopted consistent sampling, dissociation, and library preparation protocols across all sample groups. By integrating single-cell transcriptome data with single-cell chromatin accessibility data, we systematically characterized the remodeling process of the neurovascular microenvironment in tumor contexts at both expression and regulatory levels.

### 2.3 Histopathological and immunofluorescence validation

To validate the single-cell multi-omics analysis results, histological and immunofluorescence staining were performed on the remaining brain tissues. Brain tissues were fixed in 4% polyformaldehyde and processed into paraffin sections, with some samples simultaneously prepared as frozen sections. Conventional histopathology was conducted using hematoxylin-eosin staining, primarily observing characteristics such as tumor formation, tissue structure disruption, necrosis, and infiltration margins.

Immunofluorescence detection primarily focuses on vascular structure, blood-brain barrier status, changes in perivascular support cells, glial reactions, and matrix remodeling. CD31-labeled endothelial cells were used to assess microvascular density and vascular morphological changes. PDGFRβ and α-SMA were employed to detect alterations in pericytes and vascular support structures. Claudin 5 and ZO1 were utilized to evaluate the expression of blood-brain barrier-associated tight junction proteins. GFAP and Iba1 were employed to observe the activation status of astrocytes and microglia, respectively, while Collagen IV and Laminin were combined to assess extracellular matrix and basement membrane remodeling.

All sections underwent staining and image acquisition under identical conditions, followed by quantitative analysis in a blinded manner. Histological analysis was conducted to assess the protein-level expression patterns corresponding to key neurovascular states and cerebrovascular remodeling features identified through single-cell analysis by comparing changes in vascular, barrier, glial response, and matrix-related parameters across different groups.

### 2.4 Multi-omics data integration and neurovascular state annotation

After quality control, single-cell transcriptome and single-cell chromatin accessibility data were incorporated for subsequent integrated analysis. For scRNA-seq data, preprocessing was performed using Seurat (v4). Cells with fewer than 500 detected genes, fewer than 1,000 UMIs, or >10% mitochondrial transcripts were excluded. Potential doublets were identified and removed utilizing DoubletFinder. Data were normalized via the LogNormalize method, followed by principal component analysis (PCA) on the top 2,000 highly variable genes. To mitigate batch effects across biological replicates, Harmony integration was applied. Dimensionality reduction was performed using UMAP based on the top 30 principal components, and graph-based clustering was executed using the Louvain algorithm at a resolution ranging from 0.5 to 0.8 depending on the specific lineage. To mitigate the risks of pseudoreplication and integration artifacts arising from the modest sample size (n = 3 per condition), cluster stability was quantitatively assessed across individual biological replicates. Substates were exclusively retained if they were populated by cells derived from all three independent mice within a given experimental group, ensuring that the granular state definitions were not driven by data sparsity or sample-specific outliers. For scATAC-seq data, downstream processing was conducted utilizing Signac. Low-quality nuclei with a transcription start site (TSS) enrichment score <2 or unique fragments <1,000 were filtered out. Peak calling was executed using MACS2 to generate a unified peak matrix across all samples, followed by gene activity matrix construction and transcription factor motif enrichment via chromVAR. The two datasets were integrated using canonical correlation analysis (CCA) to anchor and project scATAC-seq data onto the scRNA-seq latent space. Furthermore, to delineate the continuous state transitions of endothelial and mural populations, pseudotime trajectory inference was computed using Monocle 3, rooting the trajectories in the computationally annotated homeostatic vascular clusters.

During the cell annotation process, classical marker genes, gene activity characteristics, and reported brain tissue cell lineage information were integrated to identify major cell populations including endothelial cells, pericytes and other mural cells, astrocytes, microglia or macrophages, and tumor cells. Building on this foundation, heterogeneity within each cell population was further stratified across functional dimensions such as angiogenesis, blood-brain barrier integrity, inflammatory activation, extracellular matrix remodeling, and hypoxic adaptation, thereby delineating distinct neurovascular ecosystem states. The primary cell populations and their corresponding state annotations are presented in Table 2.

**TABLE 2:** Major cell populations and annotated neurovascular ecosystem states identified by single-cell multi-omics.

Major cell population	Representative markers	Dominant molecular features	Annotated neurovascular ecosystem state	Biological interpretation
Endothelial cells	Pecam1, Cdh5, Kdr, Emcn	Vascular identity, endothelial activation, angiogenic signaling, barrier-associated programs	Homeostatic endothelial state	Maintains vascular integrity and baseline endothelial function in control brain tissues
Endothelial cells	Pecam1, Kdr, Esm1, Angpt2	Increased angiogenesis, endothelial proliferation, hypoxia-responsive transcriptional activity	Angiogenic endothelial state	Represents tumor-driven vascular expansion and abnormal endothelial remodeling
Endothelial cells	Cldn5, Ocln, Klf2, Mfsd2a	Tight junction maintenance, barrier stabilization, reduced inflammatory signaling	Barrier-maintaining vascular state	Reflects relatively preserved blood-brain barrier function
Endothelial cells	Icam1, Vcam1, Sele, Cxcl10	Inflammatory activation, leukocyte recruitment, endothelial stress response	Barrier-disrupted inflammatory state	Indicates loss of vascular homeostasis and enhanced neurovascular inflammation
Mural cells	Pdgfrb, Rgs5, Cspg4, Mcam	Perivascular support, vessel stabilization, contractile regulation	Homeostatic mural support state	Preserves vascular wall integrity and endothelial-mural coupling
Mural cells	Acta2, Tagln, Col1a1, Col3a1	Contractile remodeling, extracellular matrix deposition, perivascular stiffening	Matrix-remodeling mural state	Suggests transition toward pathological vascular support and stromal remodeling
Astrocytes	Gfap, Aqp4, Slc1a3, Aldh1l1	Perivascular homeostasis, water transport, neurovascular coupling	Homeostatic astroglial state	Supports normal neurovascular unit function and local metabolic balance
Astrocytes	Gfap, Vim, Serpina3n, Lcn2	Reactive gliosis, inflammatory signaling, stress-associated transcription	Reactive astroglial state	Reflects tumor-associated astrocytic activation and vascular niche remodeling
Microglia/macrophages	C1qa, C1qb, Aif1, Tmem119	Immune surveillance, phagocytic activity, homeostatic myeloid functions	Surveillance immune state	Represents resident microglial monitoring under relatively stable conditions
Microglia/macrophages	Aif1, Lyz2, Il1b, Apoe	Inflammatory activation, cytokine signaling, phagocytic remodeling	Inflammatory myeloid state	Indicates enhanced innate immune activation within the tumor neurovascular niche
Tumor cells	Sox2, Olig2, Pdgfra, Nestin	Proliferative signaling, lineage plasticity, stem-like features	Proliferative tumor state	Represents actively expanding tumor cell populations contributing to microenvironmental remodeling
Tumor cells	Hif1a, Vegfa, Ldha, Slc2a1	Hypoxia adaptation, metabolic reprogramming, pro-angiogenic signaling	Hypoxia-adaptive tumor niche state	Reflects tumor-driven induction of vascular remodeling under hypoxic stress
Mixed neurovascular populations	Col4a1, Col4a2, Lama4, Fn1	Basement membrane organization, extracellular matrix remodeling, vessel-associated matrix response	Perivascular matrix-remodeling state	Represents a shared remodeling program across endothelial, mural, and reactive stromal compartments

This study defines neurovascular status not solely based on single biomarker expression differences, but rather integrates cellular transcriptional characteristics, chromatin accessibility patterns, and functional program enrichment results. Through multi-omics integrated analysis, it enables more accurate differentiation between homeostatic endothelial states, angiogenic activation states, barrier-breaking inflammatory states, perivascular matrix remodeling states, and hypoxia-adaptation-related states. This analytical strategy provides a foundation for subsequent dynamic comparisons of cerebrovascular remodeling-related states, identification of key regulatory networks, and hierarchical machine learning analyses in the results section.

### 2.5 Regulatory network analysis, cell-cell communication, and machine learning modeling

After completing multi-omics integration and cell state annotation, further analysis was conducted on the regulatory programs of tumor-associated cerebrovascular remodeling. Differential expression and pathway enrichment analyses were performed based on single-cell transcriptomic data, combined with transcription factor binding site enrichment and gene activity changes from single-cell chromatin accessibility data, to identify key signaling pathways and regulatory networks associated with different neurovascular ecosystem states. Special attention was paid to functional programs such as angiogenesis, blood-brain barrier injury, inflammatory activation, extracellular matrix remodeling, and hypoxia adaptation to elucidate the primary molecular basis of cerebrovascular remodeling in a tumor context. Relevant differential pathways and regulatory programs are summarized in Table 3.

**TABLE 3:** Differential pathways and regulatory programs associated with tumor-related cerebrovascular remodeling.

Neurovascular ecosystem state	Representative pathway or regulatory program	Key genes/regulators	Enrichment score	Adjusted P value	Main biological implication
Angiogenic endothelial state	VEGF signaling	Kdr, Vegfa, Flt1, Angpt2	2.43	0.0018	Promotes endothelial activation and abnormal vascular expansion
Angiogenic endothelial state	Hypoxia response	Hif1a, Slc2a1, Ldha, Bnip3	2.17	0.0031	Supports vascular adaptation to oxygen deprivation
Barrier-disrupted inflammatory state	NF-κB signaling	Nfkb1, Rela, Icam1, Vcam1	2.36	0.0024	Drives endothelial inflammation and leukocyte recruitment
Barrier-disrupted inflammatory state	Leukocyte transendothelial migration	Sele, Ccl2, Cxcl10, Icam1	2.08	0.0042	Facilitates immune infiltration and barrier dysfunction
Matrix-remodeling mural state	Extracellular matrix organization	Col1a1, Col3a1, Fn1, Lama4	2.58	0.0012	Indicates pathological vessel support and stromal remodeling
Matrix-remodeling mural state	TGF-β signaling	Tgfb1, Tgfbr2, Smad3, Tagln	2.11	0.0048	Promotes mural activation and perivascular matrix deposition
Reactive astroglial state	Reactive gliosis program	Gfap, Vim, Serpina3n, Lcn2	2.24	0.0035	Reflects astrocytic activation in the tumor-associated niche
Reactive astroglial state	Cytokine-mediated signaling	Stat3, Il6ra, Socs3, Osmr	1.96	0.0061	Amplifies local inflammatory and stress responses
Inflammatory myeloid state	Complement activation	C1qa, C1qb, C3, Apoe	2.29	0.0029	Indicates immune remodeling and phagocytic activation
Inflammatory myeloid state	TNF signaling	Tnf, Il1b, Nfkbia, Junb	2.04	0.0053	Enhances pro-inflammatory signaling in the vascular niche
Hypoxia-adaptive tumor niche state	Glycolysis and metabolic adaptation	Ldha, Pdk1, Slc2a1, Hk2	2.31	0.0021	Supports tumor survival under hypoxic stress
Hypoxia-adaptive tumor niche state	Pro-angiogenic transcriptional program	Vegfa, Adm, Hif1a, P4ha1	2.15	0.0039	Favors vascular remodeling and niche adaptation

Intercellular communication networks were inferred using CellChat. The interaction probabilities were computed based on the law of mass action using the normalized expression of receptor-ligand pairs among major cell populations. Significant interactions were identified strictly utilizing a permutation test with 1,000 randomizations (adjusted P < 0.05). The primary analytical targets included endothelial cells, pericytes and other mural cells, astrocytes, microglia or macrophages, as well as tumor cells, to evaluate the potential strength and direction of signal interactions between different neurovascular states. Special emphasis was placed on screening ligand-receptor pairs closely associated with angiogenesis, inflammatory recruitment, matrix deposition, and perivascular niche maintenance, aiming to elucidate the multicellular synergistic communication mechanisms driving cerebrovascular remodeling in the tumor microenvironment. Key ligand-receptor interaction results are presented in Table 4.

**TABLE 4:** Key ligand-receptor interactions across neurovascular ecosystem states.

Sender cell/state	Receiver cell/state	Ligand	Receptor	Interaction score	Adjusted P value	Functional implication
Hypoxia-adaptive tumor niche state	Angiogenic endothelial state	VEGFA	KDR	0.84	0.0016	Promotes endothelial sprouting and vascular expansion
Hypoxia-adaptive tumor niche state	Matrix-remodeling mural state	PDGFB	PDGFRB	0.77	0.0034	Enhances perivascular recruitment and mural activation
Inflammatory myeloid state	Barrier-disrupted inflammatory state	TNF	TNFRSF1A	0.71	0.0041	Aggravates endothelial inflammation and barrier impairment
Inflammatory myeloid state	Reactive astroglial state	IL1B	IL1R1	0.69	0.0052	Amplifies astroglial activation in the perivascular niche
Reactive astroglial state	Endothelial cells	ANGPTL4	ITGB1	0.63	0.0085	Modulates endothelial permeability and stress adaptation
Matrix-remodeling mural state	Endothelial cells	COL1A1	ITGA1/ITGB1	0.74	0.0038	Reflects matrix-dependent endothelial remodeling
Matrix-remodeling mural state	Reactive astroglial state	TGFB1	TGFBR2	0.67	0.0063	Promotes stromal activation and matrix deposition
Endothelial cells	Inflammatory myeloid state	CCL2	CCR2	0.66	0.0071	Recruits myeloid cells into the tumor-associated vascular niche
Endothelial cells	Reactive astroglial state	SEMA3A	NRP1	0.58	0.0114	Participates in neurovascular patterning and stress signaling
Tumor cells	Reactive astroglial state	OSM	OSMR	0.61	0.0092	Supports astrocytic inflammatory reprogramming
Tumor cells	Endothelial cells	ADM	CALCRL	0.64	0.0078	Facilitates hypoxia-associated endothelial adaptation
Reactive astroglial state	Inflammatory myeloid state	CXCL12	CXCR4	0.59	0.0101	Reinforces immune retention within the perivascular niche

For machine learning modeling, feature engineering was initiated by extracting the top 100 differentially expressed genes and key differential regulatory peaks (adjusted P < 0.01, log2FC > 0.5) for each defined neurovascular state. To strictly avoid data leakage and address potential circularity originating from upstream clustering, we implemented a sample-aware biological holdout strategy using Leave-One-Sample-Out Cross-Validation (LOSO-CV). Instead of random cellular partitioning, models were trained on cells from N-1 biological replicates and evaluated on the entirely unseen holdout replicate. Furthermore, to verify that the models captured biologically non-trivial signatures rather than embedded annotation logic, label-shuffling permutation tests (1,000 iterations) were conducted to establish a null distribution of baseline performance. Supervised models—including Random Forest, XGBoost, Elastic Net Logistic Regression, and Support Vector Machines—were trained via grid search with inner cross-validation for hyperparameter tuning. Final model performance was rigorously evaluated on the independent biological holdouts using accuracy, Macro-F1 score, and area under the receiver operating characteristic curve (AUROC). Concurrently, interpretable analysis methods like SHAP were employed to rank the importance of key features, thereby identifying priority determinants driving cerebrovascular remodeling and state transitions. Model performance and key feature ranking results are presented in Table 5.

**TABLE 5:** Machine learning model performance and prioritized determinants of neurovascular state classification.

Model	Accuracy	Macro-F1 score	AUROC	Top-ranked features	Most strongly associated neurovascular states	Mean SHAP importance
Random forest	0.87	0.84	0.91	Angpt2, Cldn5, Col1a1, Pdgfrb, Vegfa	Angiogenic endothelial state, barrier-maintaining vascular state, matrix-remodeling mural state	0.124
XGBoost	0.89	0.86	0.93	Vegfa, Icam1, Fn1, Hif1a, Rgs5	Angiogenic endothelial state, barrier-disrupted inflammatory state, hypoxia-adaptive tumor niche state	0.137
Elastic Net logistic regression	0.82	0.79	0.88	Cdh5, Col4a1, Il1b, Gfap, Lama4	Barrier-maintaining vascular state, inflammatory myeloid state, reactive astroglial state	0.098
Support vector machine	0.85	0.82	0.9	Kdr, Vcam1, Tagln, Slc2a1, Cxcl10	Angiogenic endothelial state, barrier-disrupted inflammatory state, hypoxia-adaptive tumor niche state	0.112
Integrated feature ranking	​	​	​	Vegfa, Cldn5, Col1a1, Icam1, Hif1a, Pdgfrb, Fn1, Gfap	Angiogenic remodeling, barrier dysfunction, matrix remodeling, hypoxia adaptation, astroglial activation	0.129

## 3 Results

### 3.1 Construction of a single-cell atlas of the tumor-associated neurovascular microenvironment

Based on an *in situ* mouse model of brain tumors, this study first constructed a single-cell transcriptome atlas of the tumor-associated neurovascular microenvironment. Following a predefined protocol, brain tissues from control groups and early, intermediate, and advanced stages of tumor progression were collected, dissociated, and sequenced. Data integration and analysis were performed under unified quality control standards, ultimately yielding a high-quality single-cell atlas covering major neurovascular-related cell populations. The overall results demonstrate that as tumors progress, the cellular composition of brain tissues undergoes significant remodeling, particularly manifested as alterations in the proportions and states of vascular-associated cells, glial cells, immune cells, and tumor cells. These findings provide a foundation for further characterization of the neurovascular ecosystem state.

**FIGURE 1:**
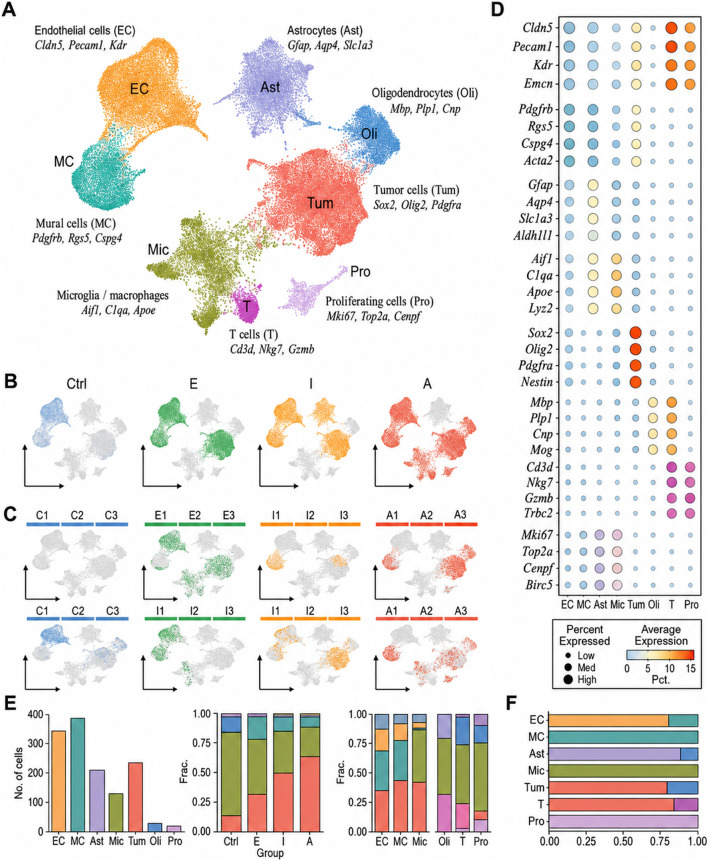
Single-cell transcriptomic atlas of the neurovascular microenvironment in tumor-bearing brain tissues. **(A)** UMAP clustering of major cell populations; **(B)** Group-wise UMAP distribution across disease progression; **(C)** Individual sample-level mini-UMAPs showing biological replicate consistency; **(D)** Marker-gene dot plot across major cell populations; **(E)** Triple composition bar plots summarizing cellular fractions; **(F)** Compact overview of atlas composition.

Figure 1A presents the global UMAP embedding of all major cell populations identified in the tumor-bearing brain tissues. The clustering pattern shows clear separation of endothelial cells, mural cells, astrocytes, microglia or macrophages, tumor cells, oligodendrocytes, T cells, and proliferating cells, indicating robust single-cell resolution and reliable annotation. Notably, endothelial and mural compartments occupy prominent and well-defined regions, supporting the notion that neurovascular elements constitute a major structural component of the tumor microenvironment.

Figure 1B illustrates the group-wise distribution of cells across Control, Early, Intermediate, and Advanced tumor stages within the same low-dimensional embedding. The overall pattern suggests progressive remodeling of the cellular landscape during tumor development. In particular, tumor-associated cell fractions become increasingly enriched in later stages, accompanied by expansion of inflammatory myeloid populations and neurovascular-associated compartments, indicating that tumor progression is coupled with dynamic reorganization of the local ecosystem.

Figure 1C presents sample-level mini-UMAPs for individual biological replicates. Despite the modest biological cohort (n = 3 per time point), the overall embedding structure and fractional distributions of major cell states were robustly conserved across replicates. We rigorously confirmed that each defined neurovascular state and substate was proportionally represented by cells from all relevant independent mice, effectively mitigating concerns regarding pseudoreplication, batch-driven integration artifacts, or sparsity-induced compositional instability.

Figure 1D provides the marker-gene dot plot used for cluster validation. Canonical lineage markers display highly restricted and coherent expression patterns across the annotated populations, confirming the accuracy of cell-type identification. Endothelial markers are concentrated in the endothelial cluster, mural markers are enriched in mural cells, glial markers are confined to astrocytes and oligodendrocytes, and immune markers are predominantly expressed in microglial or T-cell populations. This pattern reinforces the fidelity of the atlas.

Figure 1E summarizes the compositional shifts across major cell populations. The relative fractions indicate a progressive increase in tumor cells, inflammatory myeloid cells, and remodeled neurovascular populations with tumor advancement, whereas several homeostatic neural populations decline in relative abundance. These compositional trends are consistent with a transition from physiological tissue organization to a tumor-dominated and inflammation-enriched microenvironment.

Figure 1F provides an overall atlas-level composition summary. Together with the preceding panels, it confirms that the tumor-associated neurovascular microenvironment is not a static background, but a dynamically restructured multicellular system shaped by tumor growth, vascular adaptation, immune activation, and stromal remodeling.

### 3.2 Endothelial state reprogramming during tumor-associated cerebrovascular remodeling

Building upon the comprehensive single-cell atlas, this study further focuses on endothelial cell subsets to elucidate the continuous reprogramming characteristics of endothelial states during tumor-associated cerebrovascular remodeling. Computational trajectory models indicate that tumor progression is associated not solely with changes in endothelial cell abundance, but with a predicted transition from a homeostatic vascular lineage to pathological states characterized by inflammatory activation, enhanced angiogenesis, and impaired barrier function. These findings suggest that endothelial cells constitute the core responsive population in the remodeling of tumor-associated neurovascular ecosystems.

**FIGURE 2:**
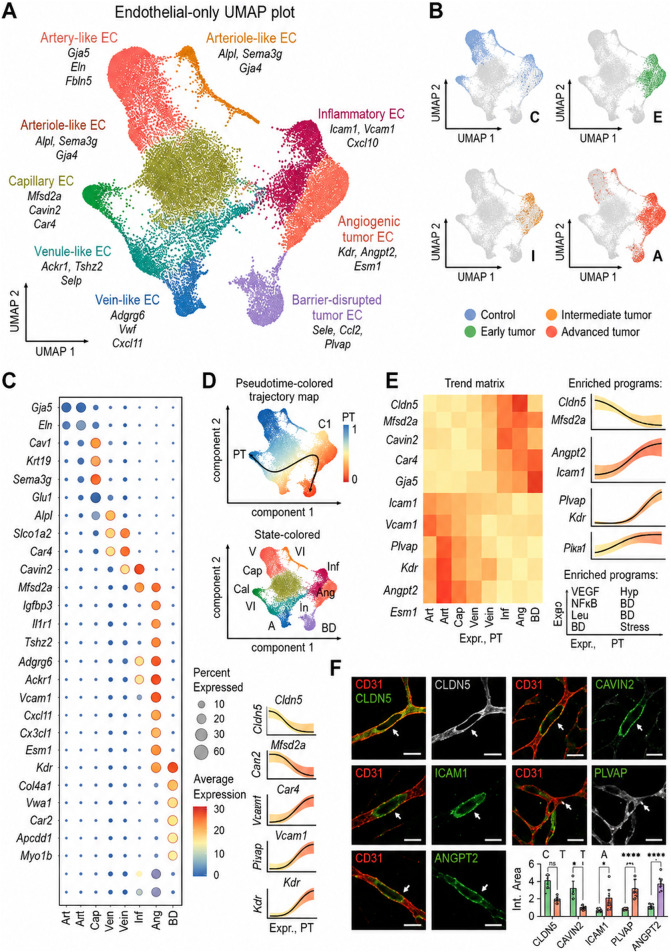
Multi-omics characterization of endothelial cell states during tumor-associated cerebrovascular remodeling. **(A)** Endothelial analysis workflow; **(B)** UMAP of endothelial states; **(C)** Marker-gene dot plot; **(D)** Pseudotime trajectory and state transition; **(E)** Dynamic expression and regulatory trends; **(F)** Multichannel immunofluorescence validation with compact quantitative summary.

Figure 2A displays the endothelial-specific UMAP after reclustering of endothelial cells from the integrated atlas. Distinct endothelial subpopulations are resolved, including artery-like, arteriole-like, capillary, venule-like, vein-like, inflammatory, angiogenic tumor-associated, and barrier-disrupted tumor-associated endothelial states. Figure 2B shows the group-wise endothelial UMAP distributions. Homeostatic endothelial states are more prominent in the control condition, whereas inflammatory, angiogenic, and barrier-disrupted endothelial populations become progressively enriched across tumor stages. Figure 2C presents the state-specific marker-gene dot plot. Artery-like and arteriole-like endothelial clusters retain characteristic vascular zonation markers, capillary endothelial cells remain enriched for barrier-associated genes, whereas inflammatory and tumor-associated endothelial states preferentially express adhesion, stress-response, permeability, and angiogenesis-related genes.

Figure 2D depicts the pseudotime trajectory of endothelial reprogramming. The trajectory suggests a continuum from homeostatic vascular identities toward inflammatory and tumor-associated pathological branches. Capillary and venule-associated endothelial states appear to serve as intermediate or transitional populations, from which inflammatory and angiogenic programs diverge.Figure 2E summarizes dynamic expression trends and regulatory programs across endothelial progression. Barrier-maintaining genes decline along the pathological trajectory, whereas genes linked to angiogenesis, inflammation, permeability, and endothelial stress rise progressively. Figure 2F provides multichannel immunofluorescence profiling of protein markers associated with the endothelial states identified computationally. Markers associated with intact vascular barrier properties are preserved in control vessels but diminished in tumor-associated vasculature, while inflammatory and permeability-related markers are elevated in pathological vascular segments.

### 3.3 Phenotypic remodeling of mural cells and emergence of matrix-associated vascular support states

Building upon the concept of endothelial cell state reprogramming, this study further analyzed the phenotypic changes of mural cells in the tumor-associated neurovascular microenvironment. The results demonstrated that as tumors progress, mural cells no longer primarily maintain vascular support and homeostatic regulatory functions, but gradually shift toward matrix remodeling, pathological vascular support, and local inflammation-related phenotypes. These findings suggest that mural cells are not only passive responders in cerebrovascular remodeling but also play a critical role in driving the reconstruction of tumor-associated vascular niches.

**FIGURE 3:**
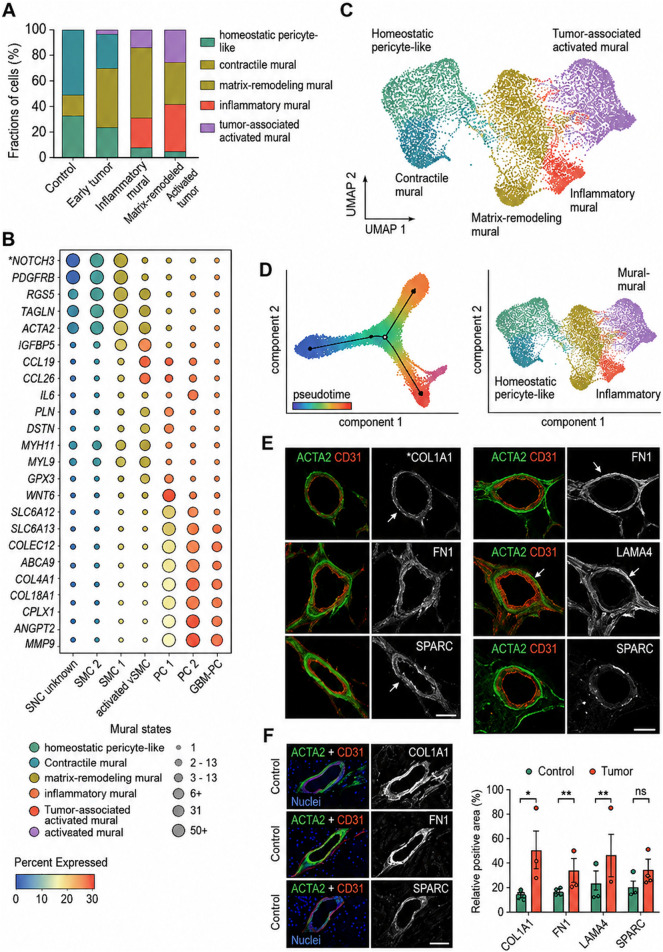
Phenotypic and regulatory remodeling of mural cells in the tumor neurovascular niche. **(A)** Stacked bar plot of mural-state fractions across groups; **(B)** Dot plot of mural markers and functional programs; **(C)** UMAP of mural subclusters; **(D)** Pseudotime trajectory of mural-state transition; **(E)** Dual-channel immunofluorescence validation; **(F)** Paired multichannel immunofluorescence with compact quantification.

Figure 3A illustrates the changes in mural cell state composition across different experimental groups. The control group predominantly exhibited steady-state pericyte-like and contractile mural states, whereas the tumor group showed significantly elevated proportions of matrix remodeling and tumor-associated activation mural states, particularly during intermediate and advanced stages. The bubble plot in Figure 3B further reveals the molecular characteristics of distinct mural states. The steady-state pericyte-like subset retained typical pericyte markers and vascular support features, the contractile subset was enriched with vascular tension regulation-related molecules, and the matrix remodeling subset exhibited markedly upregulated collagen, fibronectin, and basement membrane-associated molecules. The tumor-associated activation mural state was additionally accompanied by enhanced inflammatory and remodeling signaling. Figure 3C presents the UMAP distribution of mural cells after re-clustering. The subgroups demonstrate relatively clear boundaries while maintaining certain continuity, suggesting that mural cell state changes are not entirely discrete but rather represent progressive shifts of the same lineage under different pathological conditions.

The pseudo-time series trajectory analysis in Figure 3D further demonstrated that mural cells could transition from a steady-state pericyte-like state through a contractile phase, gradually branching into matrix remodeling and tumor-associated activation terminal states, with a small subset diverging toward inflammation-related branches. The dual-channel immunofluorescence results in Figure 3E revealed tissue-level enrichment of mural cell-associated matrix remodeling markers, consistent with the computationally predicted substate signatures. Compared to control vessels, tumor-associated vessels exhibited significantly enhanced matrix-related signals such as COL1A1, FN1, LAMA4, and SPARC, which were co-localized with ACTA2-positive supporting structures and CD31-positive vascular regions, indicating mural cells' involvement in pathological perivascular matrix deposition and scaffold remodeling. Figure 3F further compared perivascular matrix-associated signals between control and tumor tissues using multi-channel immunofluorescence and quantitative analysis. The results showed markedly increased positive areas for multiple matrix remodeling markers around vessels in the tumor group, consistent with the matrix-associated mural states identified through single-cell analysis.

### 3.4 Extracellular matrix remodeling as a shared neurovascular program in the tumor context

Building upon the aforementioned remodeling of endothelial cells and mural cells, this study further reveals that extracellular matrix remodeling is not confined to a single cell population but rather represents a shared program involving multiple cell populations within the tumor-associated neurovascular microenvironment. Integrated analysis of single-cell transcriptomics, intercellular communication, and histological validation results demonstrates that with tumor progression, basement membrane components, interstitial collagen, and matrix regulatory molecules are universally upregulated in endothelial cells, mural cells, reactive glial cells, and perivascular matrix-associated populations. These findings suggest that cerebrovascular remodeling manifests not only as vascular structural abnormalities but also accompanies significant perivascular matrix reconstruction.

**FIGURE 4:**
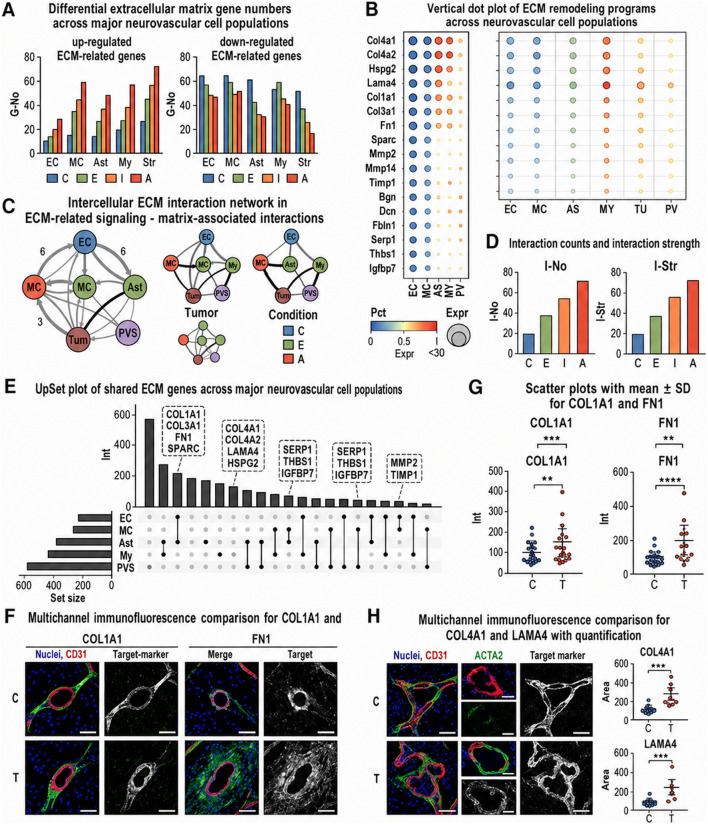
Extracellular matrix remodeling emerges as a shared feature across multiple neurovascular cell populations in the tumor context. **(A)** Grouped bar charts of differential ECM-related gene numbers; **(B)** Dot plot of ECM remodeling programs across cell populations; **(C)** ECM interaction network; **(D)** Bar charts of interaction counts and strength; **(E)** UpSet plot of shared ECM genes; **(F)** Multichannel immunofluorescence for COL1A1 and FN1; **(G)** Scatter plots with mean ± SD for COL1A1 and FN1; **(H)** Multichannel immunofluorescence and compact quantification for COL4A1 and LAMA4.

Figure 4A illustrates the variations in the number of extracellular matrix-associated differential genes across different neurovascular-related cell populations. The results demonstrate a significant increase in upregulated matrix-associated genes, particularly in the tumor group during intermediate and advanced stages, with endothelial cells, mural cells, and tumor-associated stromal populations showing the most pronounced upregulation, while downregulated genes were relatively fewer. The bubble plot in Figure 4B further reveals the distribution characteristics of extracellular matrix remodeling programs among different cell populations. Basal membrane-associated molecules such as Col4a1, Col4a2, Hspg2, and Lama4 exhibit high expression in vascular-related cells, whereas interstitial matrix molecules including Col1a1, Col3a1, Fn1, and Sparc are more prominently expressed in mural-associated states and certain perivascular stromal populations.

Figure 4C reflects the interaction networks associated with the extracellular matrix (ECM) among major cell populations. Inferred communication networks based on transcriptomic data suggest that as tumors progress, paracrine signaling potentials between endothelial cells and mural cells, mural cells and tumor cells, as well as between vascular-related populations and perivascular matrix groups significantly intensify. This pattern is suggestive of ECM remodeling functioning as a localized microenvironment network formed through multicellular synergy, rather than a single-cell autonomous event. Figure 4D summarizes the quantity and intensity of ECM-related interactions. Compared to the control group, the tumor group exhibited significantly increased numbers and intensities of ECM-associated communications, with this enhancement showing a sustained upward trend during tumor progression. The UpSet cross-set analysis in Figure 4E revealed a cohort of shared ECM-upregulated genes among different cell populations. Representative molecules including COL1A1, COL3A1, FN1, SPARC, COL4A1, COL4A2, and LAMA4 were co-enriched across multiple neurovascular-related populations, indicating that tumor-associated cerebrovascular remodeling is not dominated by a single cell type but rather involves matrix programming reorganization through the participation of multiple cell lineages.

Figure 4F demonstrates multi-channel immunofluorescence results for COL1A1 and FN1. In control brain tissues, the perivascular signals of these two matrix molecules were relatively weak, whereas in tumor tissues, their expression was significantly enhanced around CD31-positive blood vessels and ACTA2-positive supporting structures, exhibiting distinct perivascular deposition characteristics. The scatter plot in Figure 4G further quantified the expression levels of COL1A1 and FN1. Results showed that the normalized signal intensity of both proteins was significantly higher in the tumor group compared to the control group, indicating sustained enhancement of interstitial extracellular matrix (ECM) remodeling in tumor brain tissues. Figure 4H performed histological comparison and quantitative analysis of basement membrane-associated molecules COL4A1 and LAMA4. Compared to normal blood vessels, tumor tissues exhibited stronger and more irregularly distributed perivascular basement membrane signals, suggesting that tumor-associated cerebral vessels undergo abnormal matrix deposition accompanied by basement membrane structural remodeling.

### 3.5 Regional stratification of neurovascular remodeling defines niche-specific tumor ecosystems

Building upon the identification of the overall neurovascular ecosystem status, this study further analyzes tumor-associated cerebrovascular remodeling across distinct anatomical compartments. The results demonstrate that the brain tumor microenvironment is not a homogeneous structure but rather a continuous spatial hierarchy composed of the tumor core, invasive periphery, and peritumoral brain tissue. Significant differences exist among these regions in terms of cellular composition, enrichment of neurovascular states, and intensity of local remodeling. These findings suggest that tumor-associated cerebrovascular abnormalities represent not simple global alterations but rather a region-specific ecosystem reconstruction process characterized by distinct topographical distributions.

**FIGURE 5:**
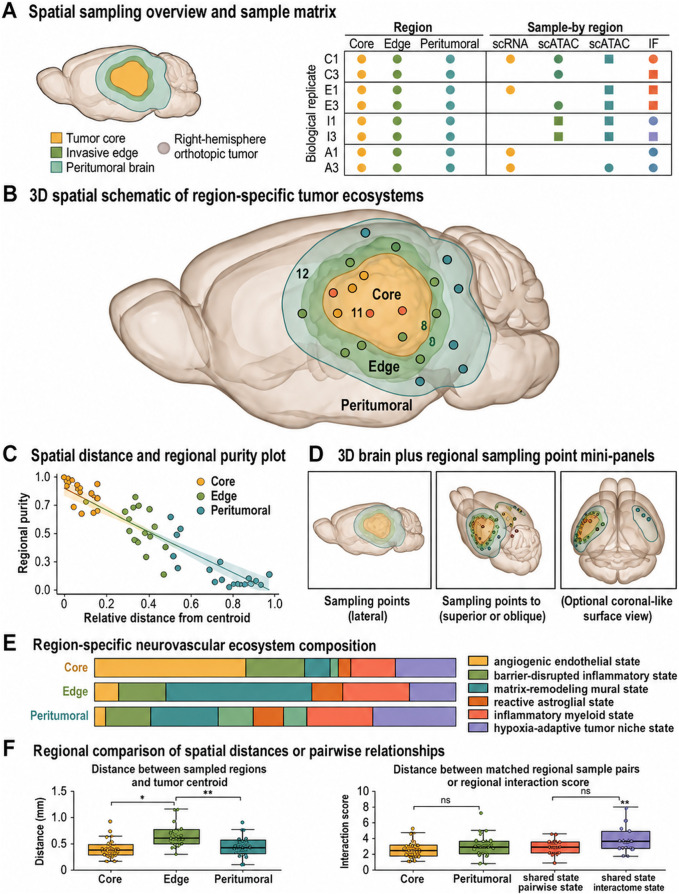
Multi-regional profiling reveals niche-specific neurovascular ecosystem remodeling in the tumor microenvironment. **(A)** Spatial sampling overview with sample-by-region matrix; **(B)** 3D brain schematic of core, edge, and peritumoral regions; **(C)** Scatter plot of relative distance from centroid versus regional purity; **(D)** 3D regional sampling-point mini-panels; **(E)** Stacked composition plot of region-specific neurovascular ecosystem states; **(F)** Boxplots of regional distance or interaction comparisons.

Figure 5A illustrates the spatial sampling strategy and sample matrix information of this study. By performing partitioned sampling of tumor core regions, invasive margins, and peritumoral brain tissues, combined with single-cell sequencing, epigenomics, and histological validation, this study enabled comparative analysis of neurovascular remodeling characteristics across different spatial regions within the same model system. This design provides a foundation for subsequent region-specific analyses and facilitates the correlation of spatial information with multi-omics results.

Figure 5B presents a three-dimensional spatial schematic of brain tissue. The results demonstrate that the tumor forms anatomically stratified regions with gradual transition from the center outward. The core region exhibits the highest tumor burden and hypoxic stress, while the peripheral zone corresponds to the tumor-invasion interface with adjacent brain tissue. The peritumoral area retains relatively intact cerebral tissue architecture. This spatial distribution suggests that distinct regions experience varying levels of metabolic, inflammatory, and mechanical stress, potentially reflecting different neurovascular ecological states.

To ensure rigorous region assignment, the anatomical boundaries were validated using a standardized metric of relative centroid distance coupled with tumor-cell purity thresholds (Figure 5C). Specifically, ‘Core’ samples were defined by a centroid distance of <0.2 and a tumor fraction >85%, while ‘Edge’ regions were identified by the visual presence of the tumor-parenchyma interface, further confirmed by regional purity gradients. This topographical stratification ensures that the observed ecosystem shifts represent stable biological differences across tumor-associated niches.

The small-scale 3D sampling point visualization from Figure 5D further confirms that sampling positioning across spatial regions exhibits good consistency and reproducibility. Sampling points in the core area are predominantly concentrated within the tumor body, while those in the peripheral zone are distributed along the tumor periphery and invasion front. The peritumoral region is located within adjacent brain tissues that have not yet been fully occupied by the tumor. These findings ensure that subsequent regional comparisons do not stem from random sampling variations but rather authentically reflect biological differences among tumor spatial niches.

Figure 5E summarizes the compositional differences in neurovascular ecosystem states across distinct spatial regions. The core region is predominantly enriched with hypoxia-adaptation-related states, angiogenesis activation states, and partial barrier dysfunction states, suggesting a bias toward metabolic stress and abnormal vasodilation in this area. The marginal region exhibits more pronounced enrichment of matrix remodeling-type mural states and invasion-supporting states, indicating its role as a critical site for tumor expansion and perivascular stent remodeling. In contrast, the peritumoral region retains relatively homeostatic vascular and glial-related states, though certain reactive alterations remain observable, suggesting exposure to tumor microenvironmental influences without complete progression to pathological terminal stages.

Figure 5F presents statistical comparisons of inter-regional distances or region-specific interaction differences. The results demonstrate significant disparities in spatial relationships and local ecological connections between the core region and the periphery, as well as between the periphery and the peritumoral area. These findings indicate that different regions do not exist independently but collectively form a tumor-associated neurovascular ecological network through continuous transitions and interactions. Notably, the periphery exhibits spatial characteristics that combine features of both the tumor core and surrounding brain tissue, further supporting its role as the frontline of tumor invasion and a critical region for neurovascular remodeling.

### 3.6 Integrated epigenomic remodeling defines regulatory transitions across neurovascular states

To further elucidate the regulatory basis between different neurovascular ecosystem states, this study integrated single-cell chromatin accessibility data with single-cell transcriptome analysis to evaluate the epigenetic foundations of tumor-associated cerebrovascular remodeling from perspectives such as local accessibility profiles, cis-regulatory element (CRE) dynamics, and transcription factor activity. The results demonstrated that transitions in neurovascular states are not merely manifested as transcriptional fluctuations but are accompanied by a series of relatively stable epigenetic remodeling processes, particularly reflected in alterations in accessibility at sites related to vascular barrier maintenance, inflammatory activation, angiogenesis, and stromal remodeling.

**FIGURE 6:**
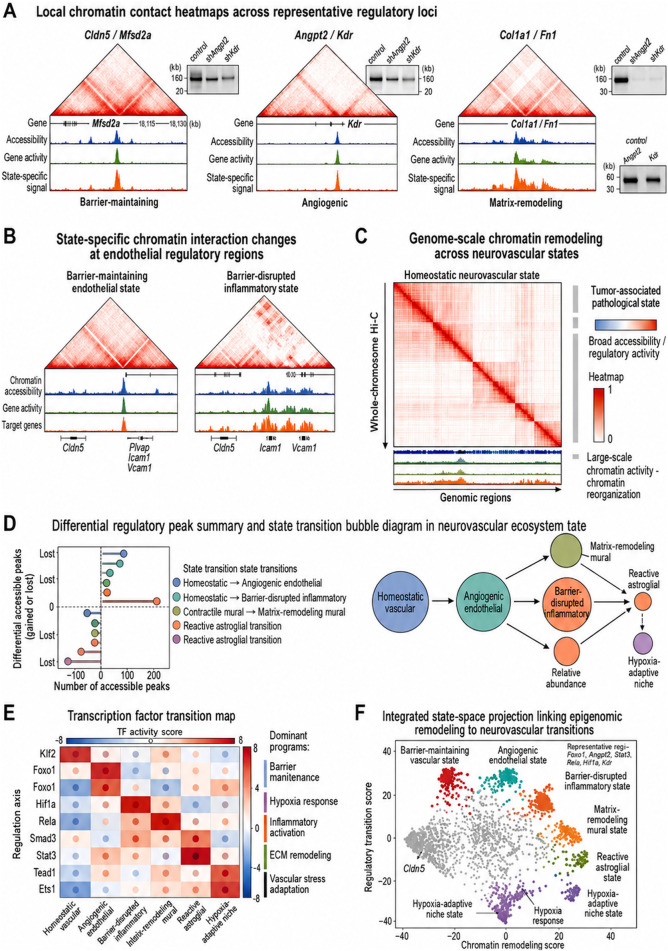
Integrated epigenomic remodeling defines regulatory transitions across neurovascular ecosystem states. **(A)** Representative chromatin accessibility landscapes with integrated genomic tracks and validation insets; **(B)** Comparison of state-specific accessibility profiles at endothelial regulatory regions; **(C)** Genome-scale chromatin remodeling heatmaps; **(D)** Differential peak summary with regulatory-state transition bubbles; **(E)** Heatmap of transcription factor activity across neurovascular states; **(F)** Integrative state-space projection linking epigenomic remodeling to regulatory transitions.

Figure 6A presents integrated chromatin accessibility signal tracks and co-accessibility loops near representative regulatory loci. The results demonstrate that in barrier-maintaining states, certain vascular homeostasis-related gene loci exhibit concentrated local contacts and well-defined regulatory structures. In contrast, in angiogenic or matrix remodeling states, loci associated with pathological remodeling show increased openness and more active local regulatory signals. Figure 6B further compares the differential accessibility of distal regulatory elements between homeostatic vascular states and pathological endothelial states within representative gene loci. The findings indicate that barrier-associated loci retain highly accessible regulatory hubs in barrier-maintaining vascular states, while these accessibility peaks are significantly attenuated in barrier-disrupted inflammatory states, accompanied by enhanced signals at loci related to inflammatory adhesion and permeability. Figure 6C provides a broader-scale visualization of chromatin remodeling characteristics across different neurovascular states. Results reveal that tumor-associated pathological states exhibit more extensive accessibility alterations and regulatory activity rearrangements in certain chromatin regions compared to homeostatic states, suggesting that neurovascular state transitions involve not only localized gene locus remodeling but also significant global epigenetic regulatory shifts.

Figure 6D summarizes the changes in the number of differential open peaks and state transition relationships during transitions between different neurovascular states. The results indicate that transitions from the steady-state vascular state to the angiogenic endothelial state, barrier-disrupted inflammatory state, and from the contractile mural state to the matrix-remodeling mural state are accompanied by the acquisition or loss of numerous differential regulatory elements. Figure 6E presents heat maps of key transcription factor activity in different neurovascular states. The results demonstrate that the steady-state vascular state is more inclined toward barrier maintenance and vascular homeostasis-related transcriptional programs, whereas the angiogenic endothelial state, barrier-disrupted inflammatory state, matrix-remodeling mural state, and hypoxia-adaptive niche state are enriched with transcriptional regulatory networks related to hypoxia response, inflammatory activation, extracellular matrix remodeling, and stress adaptation, respectively. Figure 6F provides an integrated visualization of the overall relationship between epigenetic remodeling and neurovascular state transitions. The results demonstrated relatively distinct hierarchical distributions of different neurovascular states within the integrated regulatory space. A clear separation was observed between homeostatic vascular states and pathological remodeling states, while pathological states formed a certain continuum spectrum. This suggests that they share fundamental regulatory mechanisms while retaining distinct individual tendencies.

### 3.7 Interpretable machine learning stratifies neurovascular ecosystem states and prioritizes remodeling determinants

To synthesize our multi-modal observations into a cohesive, rank-ordered hierarchy of targetable factors, this study utilized interpretable machine learning as an integrative prioritization tool, rather than a standalone predictive endpoint, to stratify tumor-associated neurovascular ecosystem states and identify key determinants associated with cerebrovascular remodeling. Evaluated under the stringent sample-aware LOSO-CV framework, the models maintained high classification performance, demonstrating that the defined neurovascular states possess robust, generalizable molecular signatures that transcend individual sample variations. Importantly, the permutation test confirmed that these AUROC values significantly outperformed the null distribution (P < 0.001), indicating that the classification learned true biological boundaries rather than circular annotation artifacts. Furthermore, this ecosystem framework provides an operational advance over conventional clustering and pathway enrichment pipelines by identifying a prioritized hierarchy of state-defining determinants that exhibit high cross-modal consistency and predictive utility. By effectively distilling complex state definitions into a quantitative molecular atlas, our approach achieves higher explanatory precision for characterizing coordinated regulatory transitions, offering a robust foundation for subsequent mechanistic interpretation.

**FIGURE 7:**
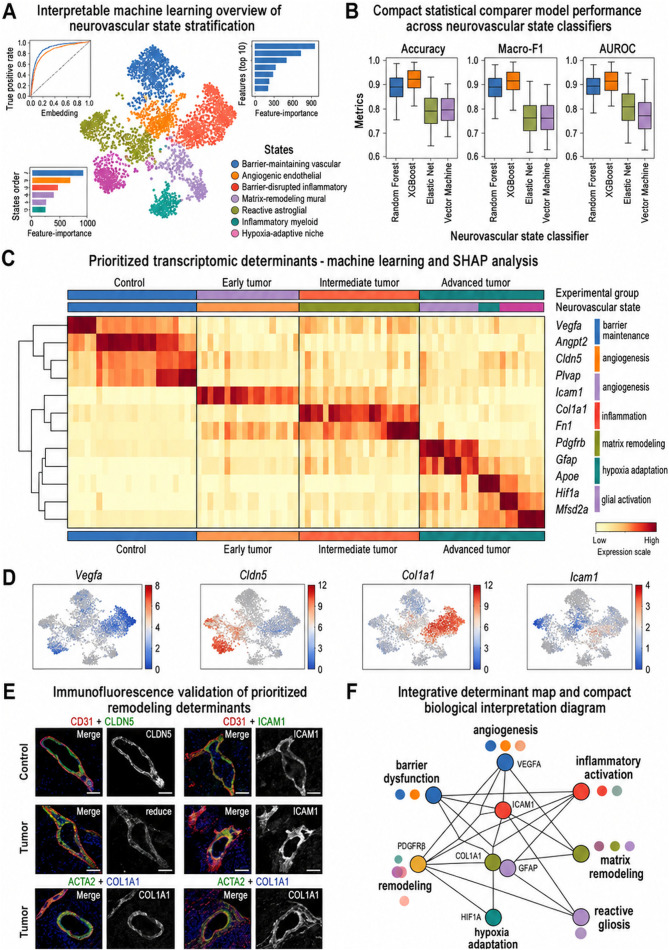
Machine learning-based stratification of transcriptomic heterogeneity across neurovascular ecosystem states. **(A)** State-embedding overview of interpretable machine learning stratification; **(B)** Boxplots comparing model performance; **(C)** Large clustered heatmap of prioritized determinants; **(D)** Feature scatter plots of key genes; **(E)** Multichannel immunofluorescence validation; **(F)** Integrative determinant-priority network.

Figure 7A demonstrates the hierarchical classification results of neurovascular ecosystem states based on interpretable machine learning. Different states form relatively distinct clustering distributions in the embedded space, with the barrier-maintaining vascular state exhibiting clear separation from pathological states such as angiogenic endothelial state, barrier-disrupted inflammatory state, and matrix-remodeling mural state, while reactive glial and hypoxia-adaptation-related states are located in continuous transitional regions. Figure 7B compares the classification performance of different machine learning models. The results indicate that random forest and XGBoost outperform elastic network logistic regression and support vector machines in metrics such as accuracy, Macro F1 score, and AUROC, suggesting that nonlinear ensemble models are more suitable for identifying complex boundaries between neurovascular states. Figure 7C presents a clustering heatmap of key remodeling determinants. The results reveal relatively distinct hierarchical patterns of molecular features representing angiogenesis, barrier maintenance, inflammatory activation, matrix remodeling, and hypoxia adaptation across different samples and states. Among these, certain barrier-maintenance-related molecules dominate in homeostatic vascular states, while angiogenesis-related, inflammatory adhesion, and matrix-associated molecules are significantly enhanced in pathological states.

Figure 7D further illustrates the distribution characteristics of several core determinants in the embedded space. The results demonstrate that some features are predominantly enriched in angiogenic endothelial or hypoxic adaptation-related regions, while others are more concentrated in barrier injury and inflammatory activation areas. Additionally, certain matrix-associated factors are primarily distributed in matrix-remodeling mural state and perivascular abnormal remodeling regions. Figure 7E presents immunofluorescence validation results for key determinants. Histological analysis reveals that homeostasis-related markers exhibit stronger expression in normal or relatively intact vessels, whereas inflammation-and matrix remodeling-associated molecules are significantly enhanced in tumor-associated abnormal vessels and perivascular regions. Figure 7F provides an integrated visualization of the relationship between key determinants and major cerebrovascular remodeling programs. The findings suggest that angiogenesis, barrier dysfunction, inflammatory activation, matrix remodeling, hypoxic adaptation, and reactive glial changes represent a coordinated molecular response, interconnected through a predicted regulatory network characterized by a set of high-priority molecules.

## 4 Discussion

This study focuses on the essence of cerebrovascular remodeling in the context of tumors. The results demonstrate that neurovascular abnormalities associated with brain tumors are not localized phenomena explainable by single-cell type or single-pathway alterations, but rather a systemic ecological reconstruction process involving endothelial cells, perivascular support cells, glial cells, myeloid cells, and tumor cells (De et al., 2026). While the individual hallmarks of the glioma microenvironment—such as abnormal angiogenesis, blood-brain barrier disruption, and matrix deposition—are widely recognized, traditional research often evaluates them as parallel but distinct events. Our findings validate these established themes but advance the field by delineating the hierarchical regulatory order and epigenetic basis of their co-occurrence. Rather than merely relabeling these phenomena, the ‘neurovascular ecosystem’ framework proposed here mathematically couples these transitions, demonstrating that barrier loss and matrix remodeling are not independent consequences of tumor growth, but synchronized outputs of a shared, ML-prioritized epigenetic program (Hovis et al., 2024). Crucially, this operational approach significantly enhances biological testability by transforming descriptive, parallel observations into a rank-ordered roadmap of regulatory dependencies, offering precise, testable hypotheses for future multi-target interventions.

Endothelial cell reprogramming stands as one of the most pivotal findings in this study. The results demonstrate that the homeostatic vascular lineage progressively deviates from its original compartmentalization characteristics under tumor conditions, transitioning toward pathological states such as inflammatory activation, abnormal angiogenesis, and barrier dysfunction (Harwood et al., 2024). This indicates that vascular abnormalities associated with brain tumors not only manifest as quantitative increases but, more critically, involve comprehensive rewritings of functional attributes and regulatory programs (Li et al., 2020). Concurrent declines in barrier-maintaining molecules coincide with elevated levels of inflammatory adhesion and permeability-related molecules, suggesting that blood-brain barrier imbalance is not a passive injury but an integral component of pathological endothelial state formation (Jain et al., 2023). These findings also elucidate why tumor regions frequently exhibit concurrent abnormal leakage, immune cell recruitment, and local metabolic dysregulation.

In addition to endothelial cells, phenotypic remodeling of mural cells is equally significant. This study reveals that pericytes and other vascular support cells do not merely contribute to vascular wall stabilization and contraction regulation, but progressively shift toward matrix deposition, perivascular scaffold reconstruction, and abnormal vascular support states during tumor progression (Hoogstrate et al., 2023). These findings suggest that mural cells are not merely accomplices in vascular abnormalities but may serve as key executors in driving the formation of pathological vascular niches. Notably, extracellular matrix-associated programs are synchronously enhanced across multiple cell populations, indicating that tumor-associated cerebrovascular remodeling is fundamentally accompanied by structural reconstruction of the perivascular microenvironment. Increased matrix deposition not only alters the local mechanical environment but may further promote tumor invasion, abnormal vascular dilation, and treatment resistance (Mathur et al., 2024).

Anatomical partitioning analysis further reinforces this understanding. The tumor core, invasive margin, and peritumoral regions do not share identical biological characteristics but correspond respectively to hypoxia and abnormal angiogenesis, stromal remodeling and invasion support, as well as transitional states that partially retain homeostasis but exhibit reactive alterations (Wälchli et al., 2024). This indicates that the tumor neurovascular ecosystem exhibits pronounced regional heterogeneity across distinct niches. Notably, the invasive margin demonstrates both pathological remodeling and transitional features of normal brain tissue, suggesting it may be the most dynamic state-transition zone and the most critical region for therapeutic intervention (Brau et al., 2025).

At the mechanistic level, this study demonstrates that neurovascular state transitions possess stable epigenetic foundations (Lemoine et al., 2025). Different states correspond to distinct chromatin accessibility patterns and transcription factor activity profiles, indicating that cerebrovascular remodeling is not a transient stress response but rather a programmed transformation accompanied by regulatory network restructuring. While supervised learning in single-cell studies risks circularity when predicting upstream unsupervised clusters, our incorporation of a strict biological holdout strategy validates the non-triviality of these states. The ability of the models to accurately classify cells in completely unseen samples confirms that these neurovascular ecosystem states represent fundamentally stable phenotypic entities rather than analytical artifacts. By leveraging interpretable machine learning, we transcended simple marker identification to quantitatively prioritize key determinants associated with angiogenesis, barrier dysfunction, inflammatory activation, matrix remodeling, and hypoxic adaptation, thereby elucidating the coordinated molecular patterns associated with vascular niche transitions.

Importantly, we acknowledge the inherent interpretative nature of our computational framework. The neurovascular states, developmental trajectories, and communication networks defined in this study represent mathematical models inferred from high-dimensional omics data rather than direct *in vivo* observations. While our parameter selections and cross-validation strategies were highly rigorous, upstream processes such as clustering resolution, pseudotime root designation, and ligand-receptor network assumptions remain sensitive to analytical choices. Consequently, the described phenomena—such as mural cells ‘acquiring’ matrix-remodeling phenotypes—should be interpreted as statistically prioritized models of cellular adaptation. Furthermore, while our sample-aware cross-validation ensured robust model performance, we recognize that the biological design of three mice per group is relatively modest for high-resolution substate partitioning. Although we explicitly verified that our defined clusters are conserved across biological replicates, the aggressive subdivision of endothelial and mural ecosystems carries inherent risks of data sparsity. Larger independent cohorts will be essential to definitively validate the compositional stability of these rare neurovascular subpopulations. Future integration with genetic lineage tracing and functional perturbation models will be necessary to definitively confirm the causal directions and absolute physical boundaries of these computationally derived ecosystem states.

## 5 Conclusion

This study leverages a murine orthotopic brain tumor model to construct a cross-disciplinary, hypothesis-generating framework for neurovascular remodeling. By utilizing single-cell transcriptomics, epigenomics, regional partitioning, and machine learning as interconnected analytical tools rather than isolated deliverables, we provide a systems-level synthesis of how multicellular ecosystems reorganize under tumor-induced stress. The results demonstrate that cerebrovascular abnormalities associated with brain tumors are not localized alterations of individual vascular components, but rather a multicellular ecological reconstruction process involving endothelial cells, mural cells, glial cells, myeloid cells, and tumor cells (Blanchette et al., 2025). Endothelial cells undergo continuous transitions from a homeostatic vascular lineage to states of inflammatory activation, enhanced angiogenesis, and barrier disruption, while mural cells gradually shift from vascular support functions to matrix deposition and abnormal perivascular support states. Concurrently, extracellular matrix remodeling serves as a shared program spanning multiple neurovascular-related cell populations, exhibiting significant spatial heterogeneity in the tumor core, invasive periphery, and peritumoral regions. Further analysis revealed that distinct neurovascular states are underpinned by clear epigenetic regulatory mechanisms, and interpretable machine learning effectively achieves state stratification and prioritizes the identification of key determinants closely associated with cerebrovascular remodeling.

## Data Availability

The raw data supporting the conclusions of this article will be made available by the authors, without undue reservation.
